# Supplementary figures and images for: Quantitative assessment of fibroblast growth factor receptor 1 expression in neurons and glia
Source: PeerJ. 2017 Apr 18;5:e3173. doi: 10.7717/peerj.3173 (PMC5398288; doi:10.7717/peerj.3173)

**GFAP****GFP****GFAP/GFP/DAPI***tgFgfr1-EGFP+*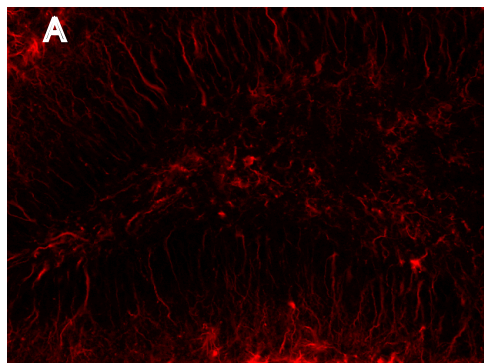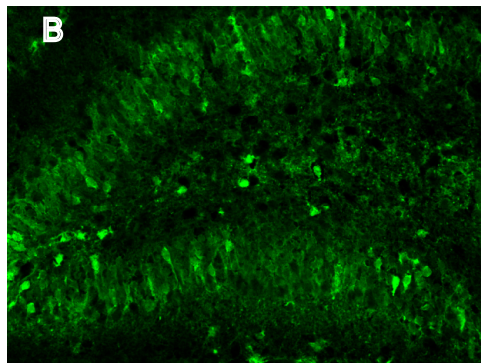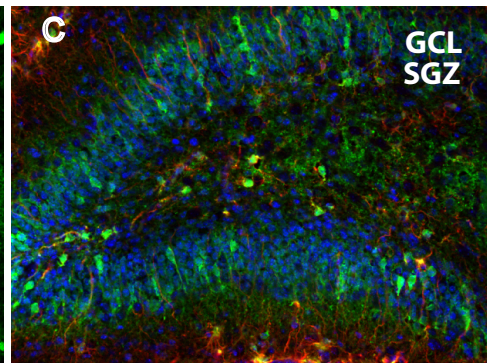

Control

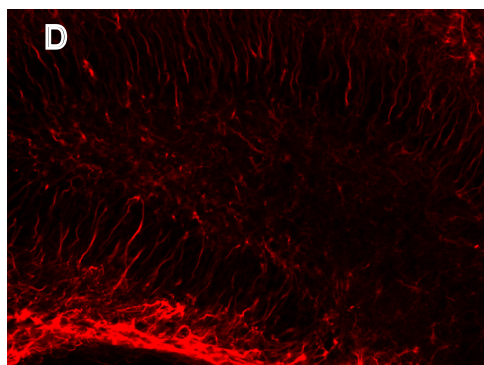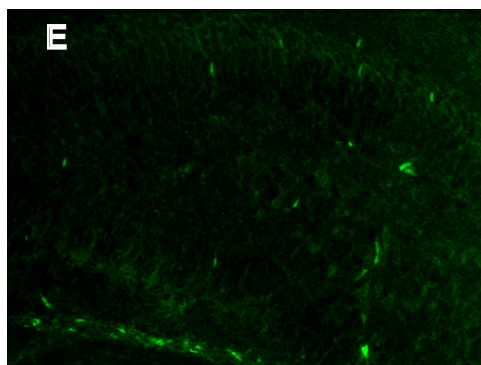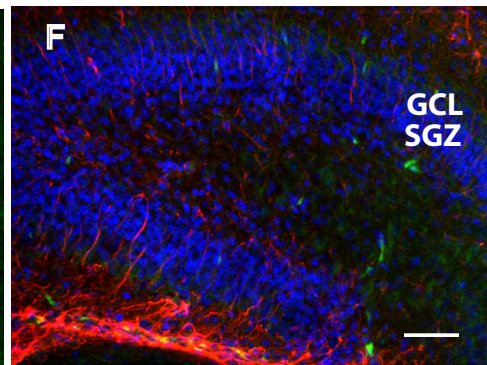*tgFgfr1-EGFP+*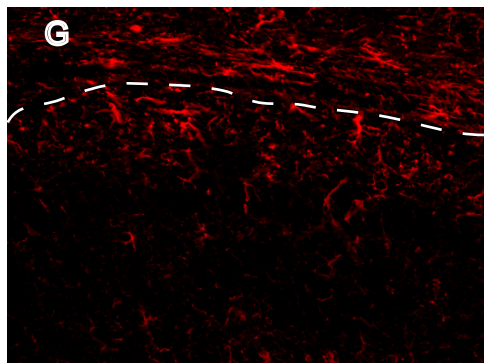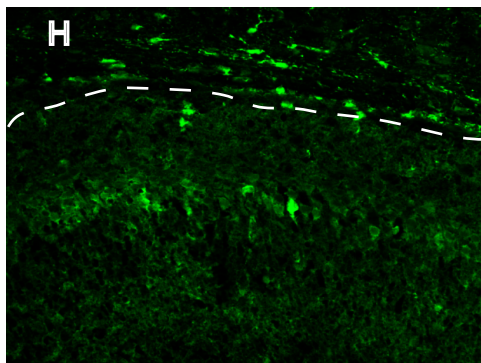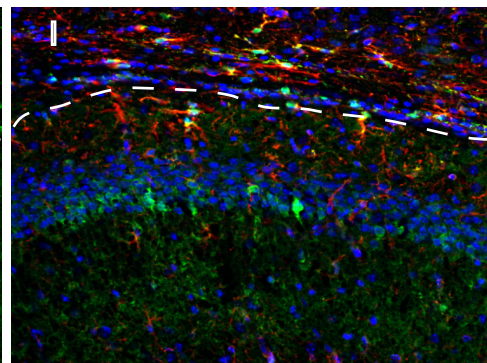

Control

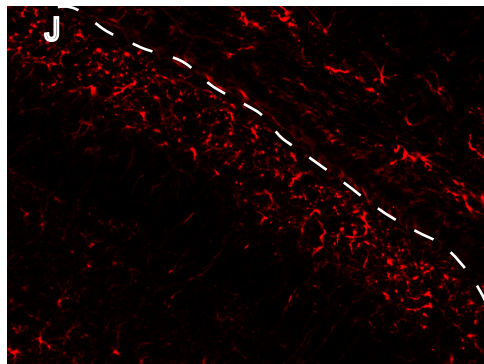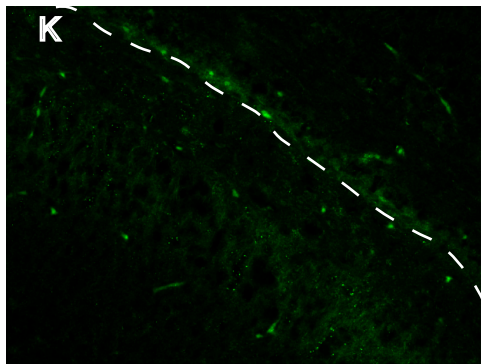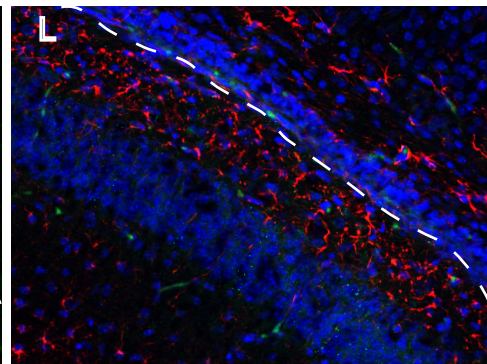

Supplement: Figure S1 — GFAP (red), and GFP immunostaining of the DG in P7 tgfgfr1-EGFP+ mice (A–C, and G–I, n = 3) and tgfgfr1-EGFP-controls (D–F, and J-L, n = 3). Comparison of the DG of the Hippocampus (A–C and D–F), and CA regions (G–I and J–L) revealed GFP+ staining in GFAP+ cells in the SGZ of the DG and in the CA region (D), as well as in white matter. DAPI, Blue channel. Dashed line indicates outer hippocampus/white matter boundary. Scale bars are 50 µm. [file peerj-05-3173-s001.pdf]

tgFgfr1-EGFP+

NeuN

GFP

NeuN/GFP/DAPI

A

SP

B

SP

C

SP

Control

D

SP

E

SP

F

SP

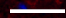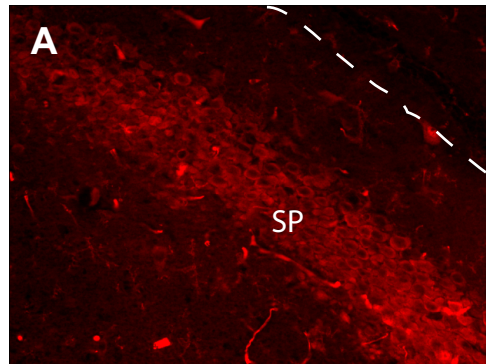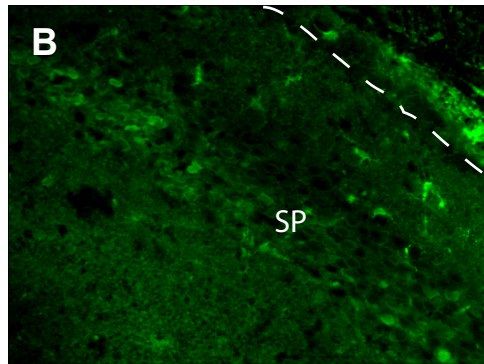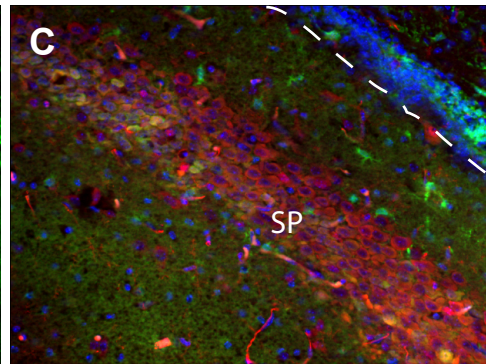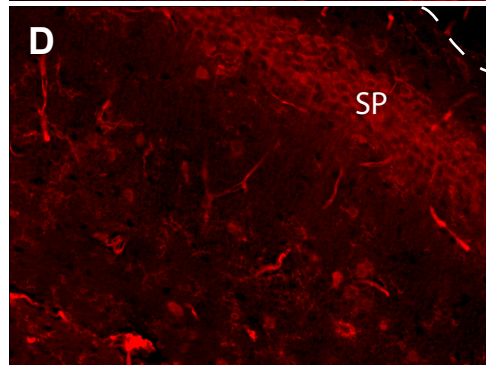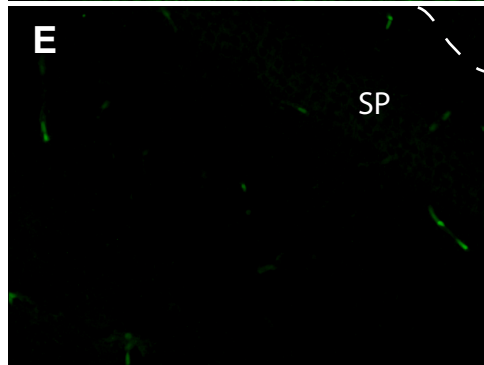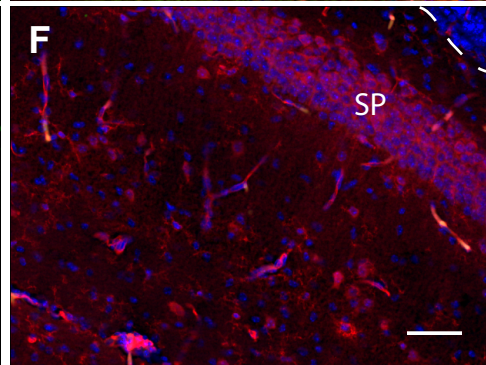

Supplement: Figure S2 — NeuN (A, D) and GFP (B, E) immunostaining of the CA region in tgfgfr1-EGFP+ mice (A–C, n = 3) and tgfgfr1-EGFP- controls (D–F, n = 3). Neun+/GFP+ staining was observed in stratum pyramidale (SP) of the CA region. Dashed line indicated outer hippocampus/white matter boundary. Scale bars are 50 µm. [file peerj-05-3173-s002.pdf]

tgFgfr1-EGFP+

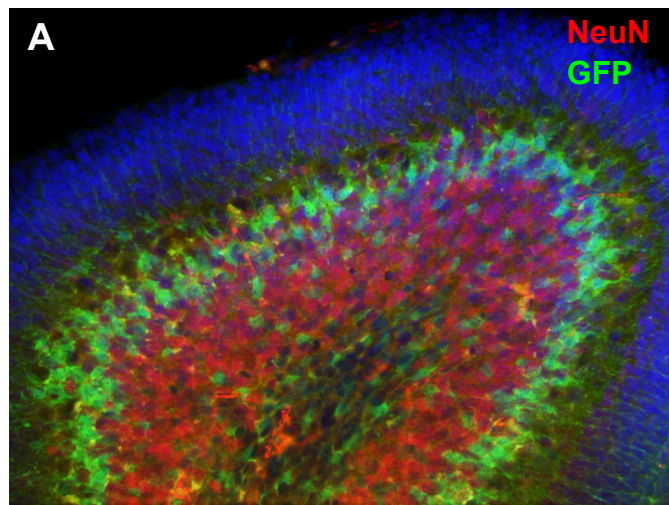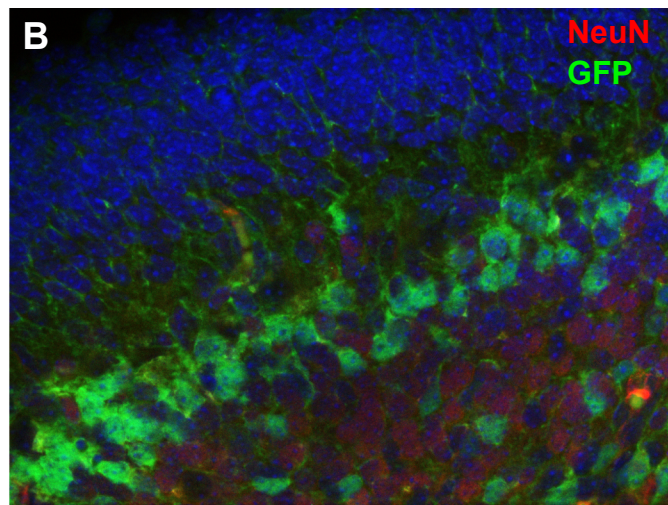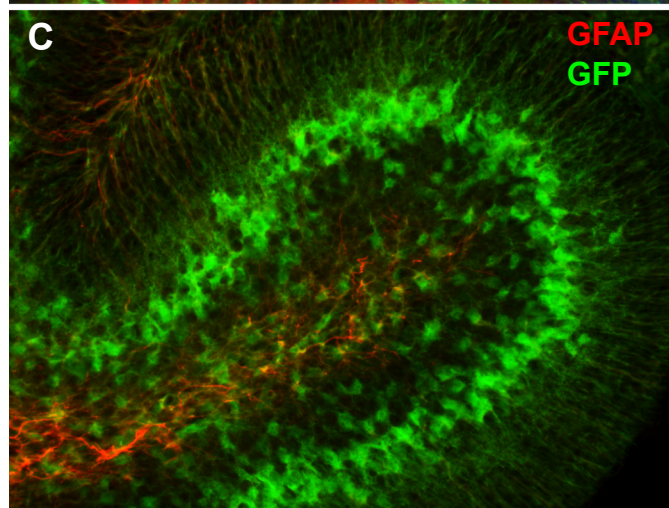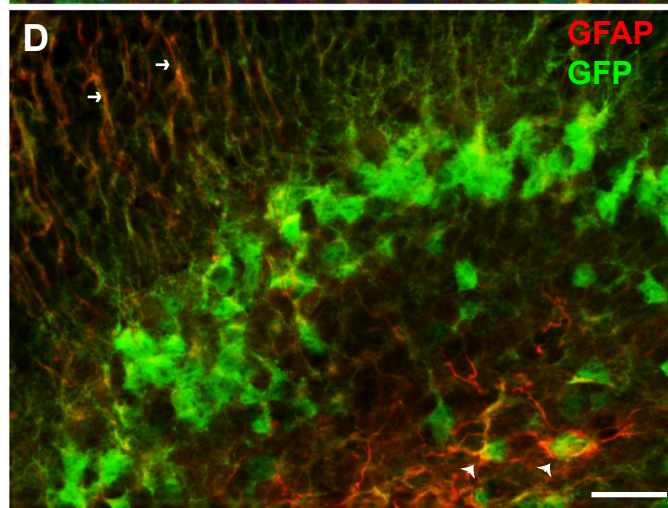

Supplement: Figure S3 — NeuN+ granule cell layer neurons did not colocalize with GFP at P7 (A, low magnification, B, high magnification). GFAP+ glia of the cerebellum do colocalize with GFP at P7 (C, low magnification, D, high magnification). Arrow heads denote double labeled cells while small arrows denote GFAP+/GFP+ glial fibers. Scale bar is 50 µm in A, C, and 25 µm in B, D. [file peerj-05-3173-s003.pdf]

Control

tgFgfr1-EGFP+

SOX2/GFP

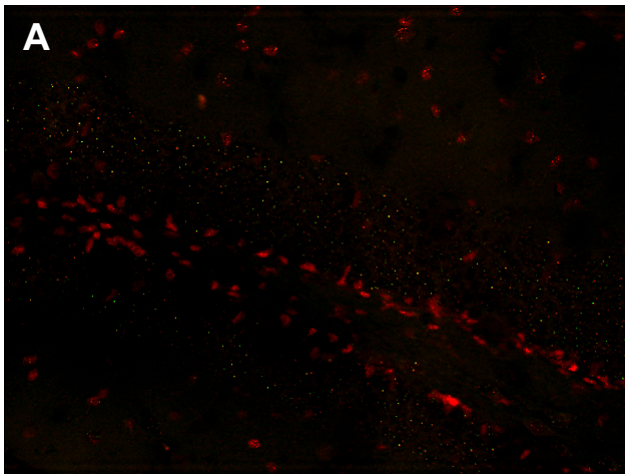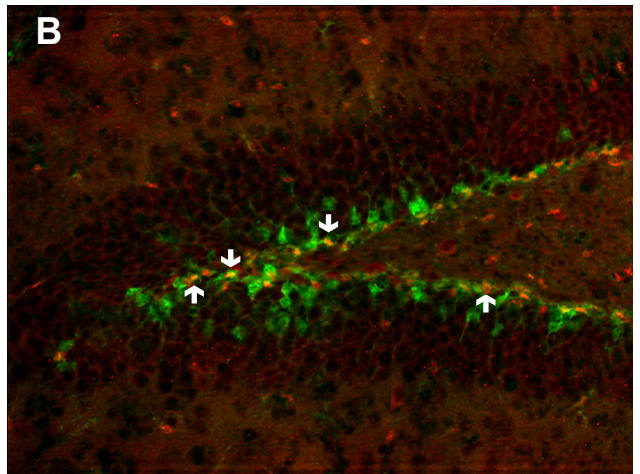

DCX/GFP

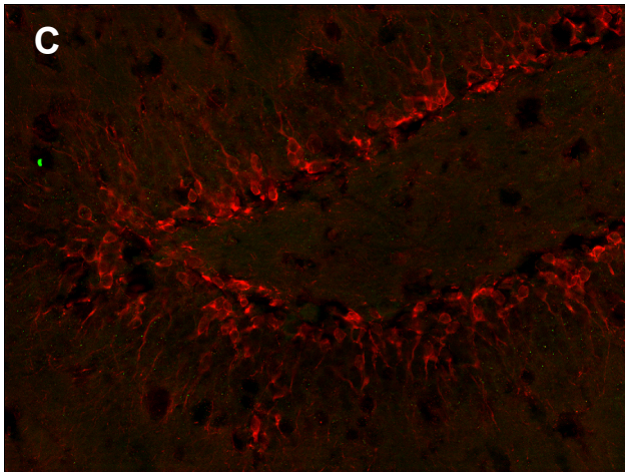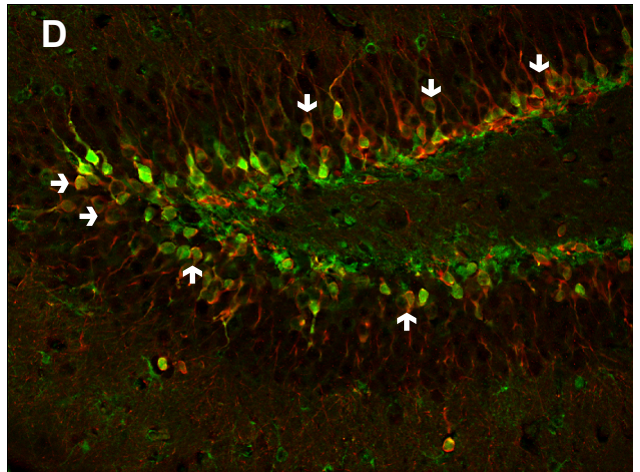

GFAP/GFP/NeuN

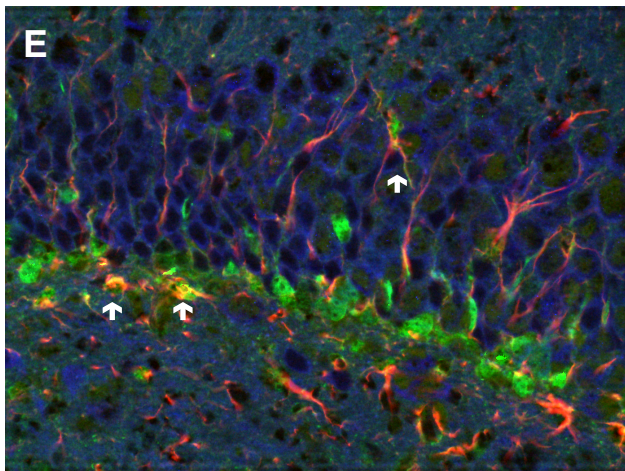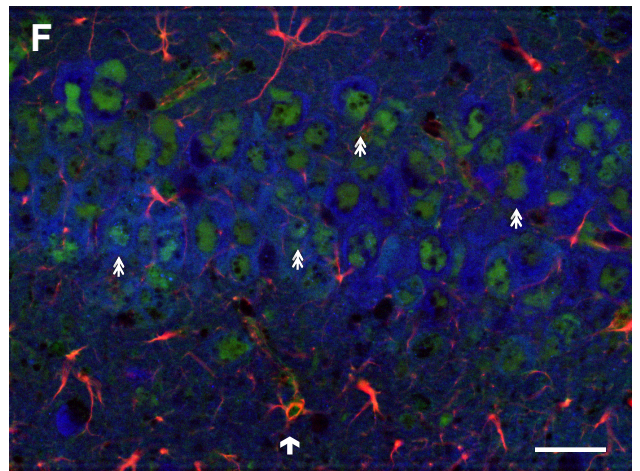

Supplement: Figure S4 — Low magnification images of SOX2 (A) and SOX2/GFP colabeling (B) in the DG of tgfgfr1-EGFP+ mice. Low magnification images of DCX (C) and DCX/GFP colabeling (D) in the DG of tgfgfr1-EGFP+ mice. High Magnification images of GFAP (red)/NeuN (blue)/GFP triple staining in the DG (E) and CA (F). Scale bar is 50 µm in A–D, and 25 µm in (E-F). [file peerj-05-3173-s004.pdf]

Control

OLIG2

GFP

DAPI

OLIG2/GFP/DAPI

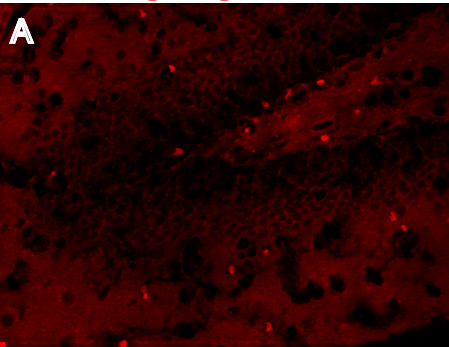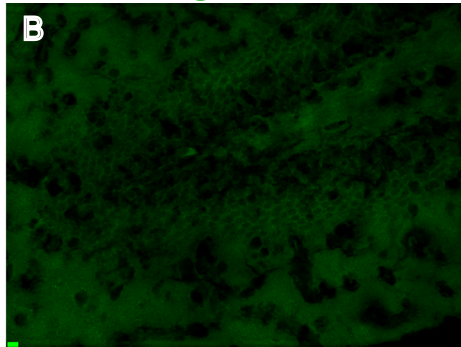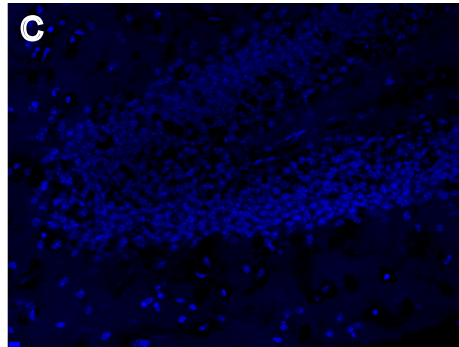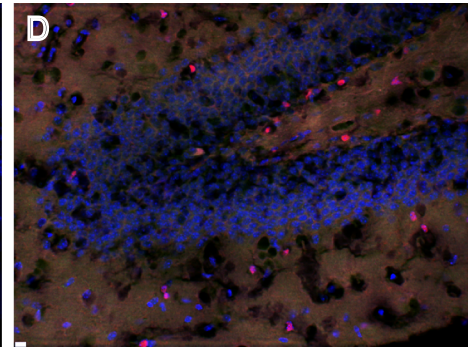

tgFgfr1-EGFP+

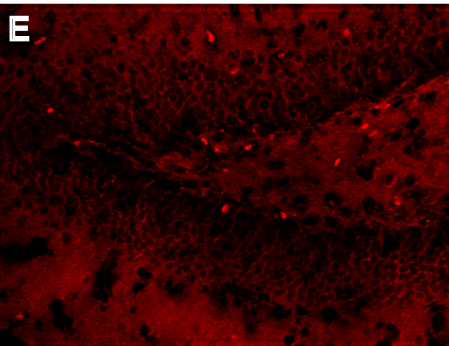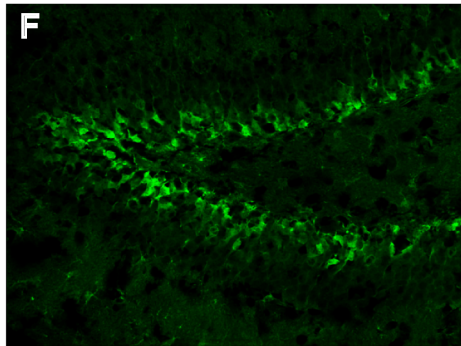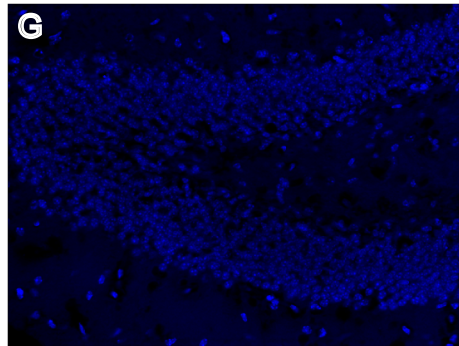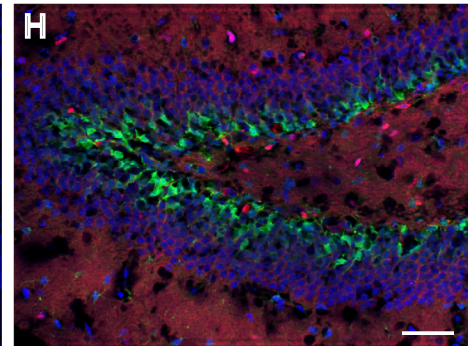

Supplement: Figure S5 — OLIG2 staining in the hippocampus of control tgfgfr1-EGFP-mice (A–D) and tgfgfr1-EGFP+ mice (E–H). Scale bar is 50 µm. [file peerj-05-3173-s005.pdf]

Control

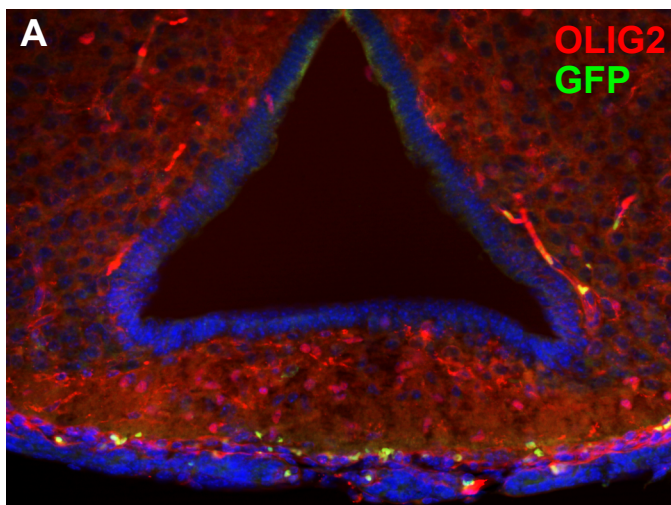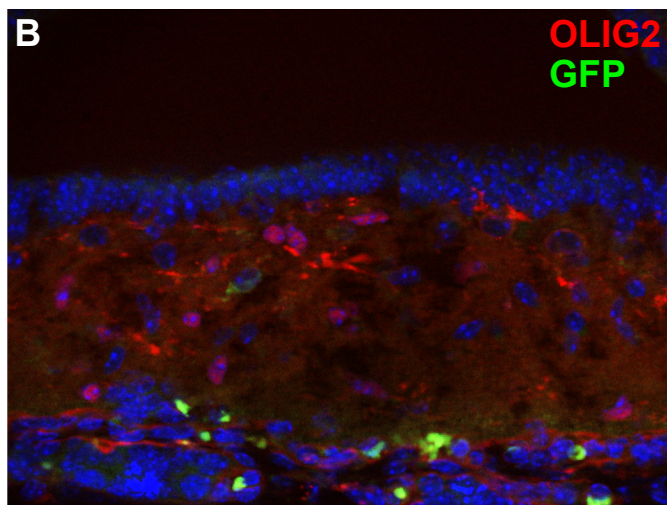

tgFgfr1-EGFP+

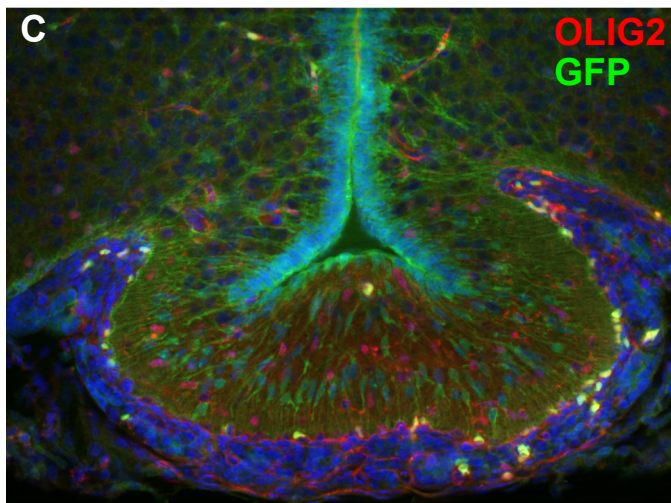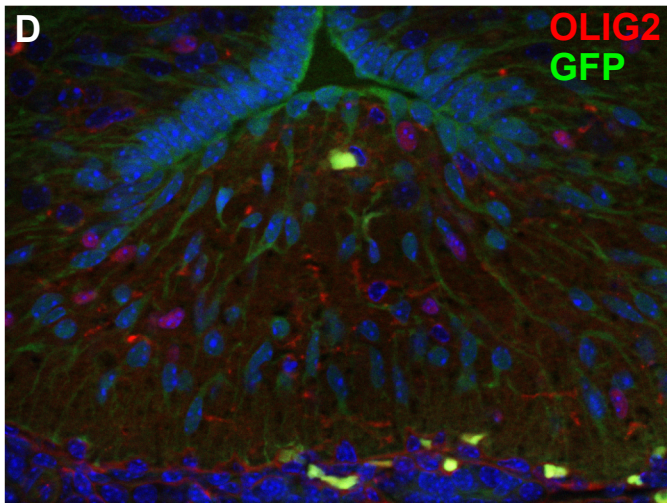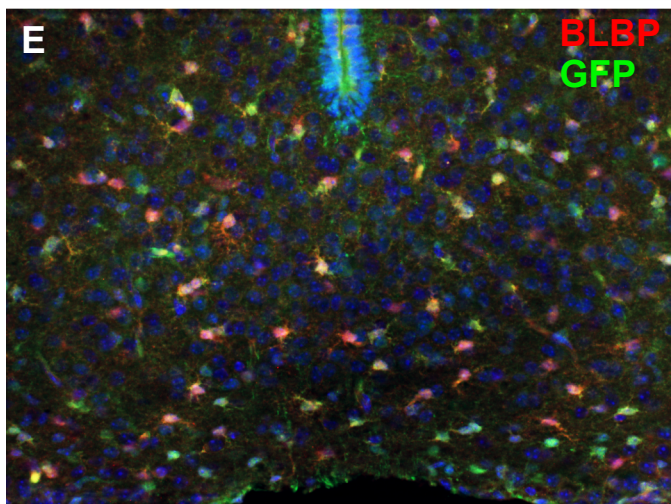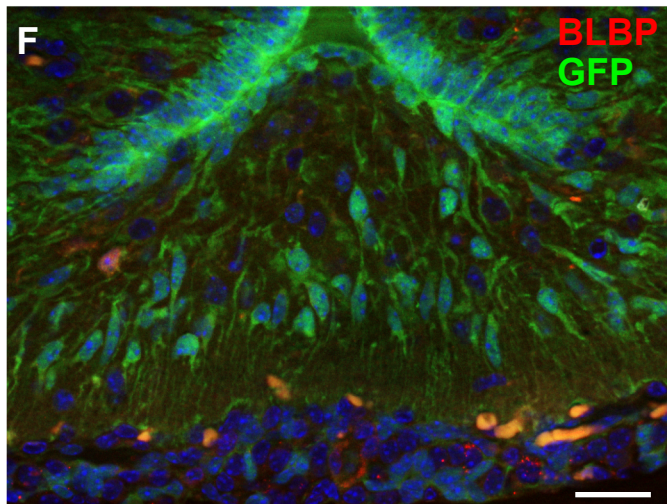

Supplement: Figure S6 — Fgfr1 promoter driven GFP in the hypothalamus of control tgfgfr1-EGFP-mice (A, B) and tgfgfr1-EGFP+ mice (C–F). OLIG2+ cells in the hypothalamus do not colocalize with Fgfr1 promoter driven GFP+ (C, D). BLBP+ cells near the third ventricle do colocolize with Fgfr1 promoter driven GFP (E), but do not colocalize with GFP in the medial eminence (F). Scale bar is 50 µm in A, C, E and 25 µm in B, D, F. [file peerj-05-3173-s006.pdf]
